# Production of ${\Sigma (1385)^{\pm }}$ and ${\Xi (1530)^{0}}$ in p–Pb collisions at ${\sqrt{s_{\mathrm{NN}}}= 5.02}$ TeV

**DOI:** 10.1140/epjc/s10052-017-4943-1

**Published:** 2017-06-13

**Authors:** D. Adamová, M. M. Aggarwal, G. Aglieri Rinella, M. Agnello, N. Agrawal, Z. Ahammed, S. Ahmad, S. U. Ahn, S. Aiola, A. Akindinov, S. N. Alam, D. S. D. Albuquerque, D. Aleksandrov, B. Alessandro, D. Alexandre, R. Alfaro Molina, A. Alici, A. Alkin, J. Alme, T. Alt, S. Altinpinar, I. Altsybeev, C. Alves Garcia Prado, M. An, C. Andrei, H. A. Andrews, A. Andronic, V. Anguelov, C. Anson, T. Antičić, F. Antinori, P. Antonioli, R. Anwar, L. Aphecetche, H. Appelshäuser, S. Arcelli, R. Arnaldi, O. W. Arnold, I. C. Arsene, M. Arslandok, B. Audurier, A. Augustinus, R. Averbeck, M. D. Azmi, A. Badalà, Y. W. Baek, S. Bagnasco, R. Bailhache, R. Bala, A. Baldisseri, M. Ball, R. C. Baral, A. M. Barbano, R. Barbera, F. Barile, L. Barioglio, G. G. Barnaföldi, L. S. Barnby, V. Barret, P. Bartalini, K. Barth, J. Bartke, E. Bartsch, M. Basile, N. Bastid, S. Basu, B. Bathen, G. Batigne, A. Batista Camejo, B. Batyunya, P. C. Batzing, I. G. Bearden, H. Beck, C. Bedda, N. K. Behera, I. Belikov, F. Bellini, H. Bello Martinez, R. Bellwied, L. G. E. Beltran, V. Belyaev, G. Bencedi, S. Beole, A. Bercuci, Y. Berdnikov, D. Berenyi, R. A. Bertens, D. Berzano, L. Betev, A. Bhasin, I. R. Bhat, A. K. Bhati, B. Bhattacharjee, J. Bhom, L. Bianchi, N. Bianchi, C. Bianchin, J. Bielčík, J. Bielčíková, A. Bilandzic, G. Biro, R. Biswas, S. Biswas, J. T. Blair, D. Blau, C. Blume, G. Boca, F. Bock, A. Bogdanov, L. Boldizsár, M. Bombara, G. Bonomi, M. Bonora, J. Book, H. Borel, A. Borissov, M. Borri, E. Botta, C. Bourjau, P. Braun-Munzinger, M. Bregant, T. A. Broker, T. A. Browning, M. Broz, E. J. Brucken, E. Bruna, G. E. Bruno, D. Budnikov, H. Buesching, S. Bufalino, P. Buhler, S. A. I. Buitron, P. Buncic, O. Busch, Z. Buthelezi, J. B. Butt, J. T. Buxton, J. Cabala, D. Caffarri, H. Caines, A. Caliva, E. Calvo Villar, P. Camerini, A. A. Capon, F. Carena, W. Carena, F. Carnesecchi, J. Castillo Castellanos, A. J. Castro, E. A. R. Casula, C. Ceballos Sanchez, P. Cerello, B. Chang, S. Chapeland, M. Chartier, J. L. Charvet, S. Chattopadhyay, S. Chattopadhyay, A. Chauvin, M. Cherney, C. Cheshkov, B. Cheynis, V. Chibante Barroso, D. D. Chinellato, S. Cho, P. Chochula, K. Choi, M. Chojnacki, S. Choudhury, P. Christakoglou, C. H. Christensen, P. Christiansen, T. Chujo, S. U. Chung, C. Cicalo, L. Cifarelli, F. Cindolo, J. Cleymans, F. Colamaria, D. Colella, A. Collu, M. Colocci, G. Conesa Balbastre, Z. Conesa del Valle, M. E. Connors, J. G. Contreras, T. M. Cormier, Y. Corrales Morales, I. Cortés Maldonado, P. Cortese, M. R. Cosentino, F. Costa, S. Costanza, J. Crkovská, P. Crochet, E. Cuautle, L. Cunqueiro, T. Dahms, A. Dainese, M. C. Danisch, A. Danu, D. Das, I. Das, S. Das, A. Dash, S. Dash, S. De, A. De Caro, G. de Cataldo, C. de Conti, J. de Cuveland, A. De Falco, D. De Gruttola, N. De Marco, S. De Pasquale, R. D. De Souza, H. F. Degenhardt, A. Deisting, A. Deloff, C. Deplano, P. Dhankher, D. Di Bari, A. Di Mauro, P. Di Nezza, B. Di Ruzza, M. A. Diaz Corchero, T. Dietel, P. Dillenseger, R. Divià, Ø. Djuvsland, A. Dobrin, D. Domenicis Gimenez, B. Dönigus, O. Dordic, T. Drozhzhova, A. K. Dubey, A. Dubla, L. Ducroux, A. K. Duggal, P. Dupieux, R. J. Ehlers, D. Elia, E. Endress, H. Engel, E. Epple, B. Erazmus, F. Erhardt, B. Espagnon, S. Esumi, G. Eulisse, J. Eum, D. Evans, S. Evdokimov, L. Fabbietti, D. Fabris, J. Faivre, A. Fantoni, M. Fasel, L. Feldkamp, A. Feliciello, G. Feofilov, J. Ferencei, A. Fernández Téllez, E. G. Ferreiro, A. Ferretti, A. Festanti, V. J. G. Feuillard, J. Figiel, M. A. S. Figueredo, S. Filchagin, D. Finogeev, F. M. Fionda, E. M. Fiore, M. Floris, S. Foertsch, P. Foka, S. Fokin, E. Fragiacomo, A. Francescon, A. Francisco, U. Frankenfeld, G. G. Fronze, U. Fuchs, C. Furget, A. Furs, M. Fusco Girard, J. J. Gaardhøje, M. Gagliardi, A. M. Gago, K. Gajdosova, M. Gallio, C. D. Galvan, D. R. Gangadharan, P. Ganoti, C. Gao, C. Garabatos, E. Garcia-Solis, K. Garg, P. Garg, C. Gargiulo, P. Gasik, E. F. Gauger, M. B. Gay Ducati, M. Germain, P. Ghosh, S. K. Ghosh, P. Gianotti, P. Giubellino, P. Giubilato, E. Gladysz-Dziadus, P. Glässel, D. M. Goméz Coral, A. Gomez Ramirez, A. S. Gonzalez, V. Gonzalez, P. González-Zamora, S. Gorbunov, L. Görlich, S. Gotovac, V. Grabski, L. K. Graczykowski, K. L. Graham, L. Greiner, A. Grelli, C. Grigoras, V. Grigoriev, A. Grigoryan, S. Grigoryan, N. Grion, J. M. Gronefeld, F. Grosa, J. F. Grosse-Oetringhaus, R. Grosso, L. Gruber, F. R. Grull, F. Guber, R. Guernane, B. Guerzoni, K. Gulbrandsen, T. Gunji, A. Gupta, R. Gupta, I. B. Guzman, R. Haake, C. Hadjidakis, H. Hamagaki, G. Hamar, J. C. Hamon, J. W. Harris, A. Harton, D. Hatzifotiadou, S. Hayashi, S. T. Heckel, E. Hellbär, H. Helstrup, A. Herghelegiu, G. Herrera Corral, F. Herrmann, B. A. Hess, K. F. Hetland, H. Hillemanns, B. Hippolyte, J. Hladky, D. Horak, R. Hosokawa, P. Hristov, C. Hughes, T. J. Humanic, N. Hussain, T. Hussain, D. Hutter, D. S. Hwang, R. Ilkaev, M. Inaba, M. Ippolitov, M. Irfan, V. Isakov, M. S. Islam, M. Ivanov, V. Ivanov, V. Izucheev, B. Jacak, N. Jacazio, P. M. Jacobs, M. B. Jadhav, S. Jadlovska, J. Jadlovsky, C. Jahnke, M. J. Jakubowska, M. A. Janik, P. H. S. Y. Jayarathna, C. Jena, S. Jena, M. Jercic, R. T. Jimenez Bustamante, P. G. Jones, A. Jusko, P. Kalinak, A. Kalweit, J. H. Kang, V. Kaplin, S. Kar, A. Karasu Uysal, O. Karavichev, T. Karavicheva, L. Karayan, E. Karpechev, U. Kebschull, R. Keidel, D. L. D. Keijdener, M. Keil, B. Ketzer, M. Mohisin Khan, P. Khan, S. A. Khan, A. Khanzadeev, Y. Kharlov, A. Khatun, A. Khuntia, M. M. Kielbowicz, B. Kileng, D. W. Kim, D. J. Kim, D. Kim, H. Kim, J. S. Kim, J. Kim, M. Kim, M. Kim, S. Kim, T. Kim, S. Kirsch, I. Kisel, S. Kiselev, A. Kisiel, G. Kiss, J. L. Klay, C. Klein, J. Klein, C. Klein-Bösing, S. Klewin, A. Kluge, M. L. Knichel, A. G. Knospe, C. Kobdaj, M. Kofarago, T. Kollegger, A. Kolojvari, V. Kondratiev, N. Kondratyeva, E. Kondratyuk, A. Konevskikh, M. Kopcik, M. Kour, C. Kouzinopoulos, O. Kovalenko, V. Kovalenko, M. Kowalski, G. Koyithatta Meethaleveedu, I. Králik, A. Kravčáková, M. Krivda, F. Krizek, E. Kryshen, M. Krzewicki, A. M. Kubera, V. Kučera, C. Kuhn, P. G. Kuijer, A. Kumar, J. Kumar, L. Kumar, S. Kumar, S. Kundu, P. Kurashvili, A. Kurepin, A. B. Kurepin, A. Kuryakin, S. Kushpil, M. J. Kweon, Y. Kwon, S. L. La Pointe, P. La Rocca, C. Lagana Fernandes, I. Lakomov, R. Langoy, K. Lapidus, C. Lara, A. Lardeux, A. Lattuca, E. Laudi, R. Lavicka, L. Lazaridis, R. Lea, L. Leardini, S. Lee, F. Lehas, S. Lehner, J. Lehrbach, R. C. Lemmon, V. Lenti, E. Leogrande, I. León Monzón, P. Lévai, S. Li, X. Li, J. Lien, R. Lietava, S. Lindal, V. Lindenstruth, C. Lippmann, M. A. Lisa, V. Litichevskyi, H. M. Ljunggren, W. J. Llope, D. F. Lodato, P. I. Loenne, V. Loginov, C. Loizides, P. Loncar, X. Lopez, E. López Torres, A. Lowe, P. Luettig, M. Lunardon, G. Luparello, M. Lupi, T. H. Lutz, A. Maevskaya, M. Mager, S. Mahajan, S. M. Mahmood, A. Maire, R. D. Majka, M. Malaev, I. Maldonado Cervantes, L. Malinina, D. Mal’Kevich, P. Malzacher, A. Mamonov, V. Manko, F. Manso, V. Manzari, Y. Mao, M. Marchisone, J. Mareš, G. V. Margagliotti, A. Margotti, J. Margutti, A. Marín, C. Markert, M. Marquard, N. A. Martin, P. Martinengo, J. A. L. Martinez, M. I. Martínez, G. Martínez García, M. Martinez Pedreira, A. Mas, S. Masciocchi, M. Masera, A. Masoni, A. Mastroserio, A. M. Mathis, A. Matyja, C. Mayer, J. Mazer, M. Mazzilli, M. A. Mazzoni, F. Meddi, Y. Melikyan, A. Menchaca-Rocha, E. Meninno, J. Mercado Pérez, M. Meres, S. Mhlanga, Y. Miake, M. M. Mieskolainen, D. Mihaylov, K. Mikhaylov, L. Milano, J. Milosevic, A. Mischke, A. N. Mishra, D. Miśkowiec, J. Mitra, C. M. Mitu, N. Mohammadi, B. Mohanty, E. Montes, D. A. Moreira De Godoy, L. A. P. Moreno, S. Moretto, A. Morreale, A. Morsch, V. Muccifora, E. Mudnic, D. Mühlheim, S. Muhuri, M. Mukherjee, J. D. Mulligan, M. G. Munhoz, K. Münning, R. H. Munzer, H. Murakami, S. Murray, L. Musa, J. Musinsky, C. J. Myers, B. Naik, R. Nair, B. K. Nandi, R. Nania, E. Nappi, M. U. Naru, H. Natal da Luz, C. Nattrass, S. R. Navarro, K. Nayak, R. Nayak, T. K. Nayak, S. Nazarenko, A. Nedosekin, R. A. Negrao De Oliveira, L. Nellen, S. V. Nesbo, F. Ng, M. Nicassio, M. Niculescu, J. Niedziela, B. S. Nielsen, S. Nikolaev, S. Nikulin, V. Nikulin, F. Noferini, P. Nomokonov, G. Nooren, J. C. C. Noris, J. Norman, A. Nyanin, J. Nystrand, H. Oeschler, S. Oh, A. Ohlson, T. Okubo, L. Olah, J. Oleniacz, A. C. Oliveira Da Silva, M. H. Oliver, J. Onderwaater, C. Oppedisano, R. Orava, M. Oravec, A. Ortiz Velasquez, A. Oskarsson, J. Otwinowski, K. Oyama, M. Ozdemir, Y. Pachmayer, V. Pacik, D. Pagano, P. Pagano, G. Paić, S. K. Pal, P. Palni, J. Pan, A. K. Pandey, S. Panebianco, V. Papikyan, G. S. Pappalardo, P. Pareek, J. Park, W. J. Park, S. Parmar, A. Passfeld, S. P. Pathak, V. Paticchio, R. N. Patra, B. Paul, H. Pei, T. Peitzmann, X. Peng, L. G. Pereira, H. Pereira Da Costa, D. Peresunko, E. Perez Lezama, V. Peskov, Y. Pestov, V. Petráček, V. Petrov, M. Petrovici, C. Petta, R. P. Pezzi, S. Piano, M. Pikna, P. Pillot, L. O. D. L. Pimentel, O. Pinazza, L. Pinsky, D. B. Piyarathna, M. Płoskoń, M. Planinic, J. Pluta, S. Pochybova, P. L. M. Podesta-Lerma, M. G. Poghosyan, B. Polichtchouk, N. Poljak, W. Poonsawat, A. Pop, H. Poppenborg, S. Porteboeuf-Houssais, J. Porter, J. Pospisil, V. Pozdniakov, S. K. Prasad, R. Preghenella, F. Prino, C. A. Pruneau, I. Pshenichnov, M. Puccio, G. Puddu, P. Pujahari, V. Punin, J. Putschke, H. Qvigstad, A. Rachevski, S. Raha, S. Rajput, J. Rak, A. Rakotozafindrabe, L. Ramello, F. Rami, D. B. Rana, R. Raniwala, S. Raniwala, S. S. Räsänen, B. T. Rascanu, D. Rathee, V. Ratza, I. Ravasenga, K. F. Read, K. Redlich, A. Rehman, P. Reichelt, F. Reidt, X. Ren, R. Renfordt, A. R. Reolon, A. Reshetin, K. Reygers, V. Riabov, R. A. Ricci, T. Richert, M. Richter, P. Riedler, W. Riegler, F. Riggi, C. Ristea, M. Rodríguez Cahuantzi, K. Røed, E. Rogochaya, D. Rohr, D. Röhrich, P. S. Rokita, F. Ronchetti, L. Ronflette, P. Rosnet, A. Rossi, A. Rotondi, F. Roukoutakis, A. Roy, C. Roy, P. Roy, A. J. Rubio Montero, R. Rui, R. Russo, A. Rustamov, E. Ryabinkin, Y. Ryabov, A. Rybicki, S. Saarinen, S. Sadhu, S. Sadovsky, K. Šafařík, S. K. Saha, B. Sahlmuller, B. Sahoo, P. Sahoo, R. Sahoo, S. Sahoo, P. K. Sahu, J. Saini, S. Sakai, M. A. Saleh, J. Salzwedel, S. Sambyal, V. Samsonov, A. Sandoval, D. Sarkar, N. Sarkar, P. Sarma, M. H. P. Sas, E. Scapparone, F. Scarlassara, R. P. Scharenberg, H. S. Scheid, C. Schiaua, R. Schicker, C. Schmidt, H. R. Schmidt, M. O. Schmidt, M. Schmidt, J. Schukraft, Y. Schutz, K. Schwarz, K. Schweda, G. Scioli, E. Scomparin, R. Scott, M. Šefčík, J. E. Seger, Y. Sekiguchi, D. Sekihata, I. Selyuzhenkov, K. Senosi, S. Senyukov, E. Serradilla, P. Sett, A. Sevcenco, A. Shabanov, A. Shabetai, O. Shadura, R. Shahoyan, A. Shangaraev, A. Sharma, A. Sharma, M. Sharma, M. Sharma, N. Sharma, A. I. Sheikh, K. Shigaki, Q. Shou, K. Shtejer, Y. Sibiriak, S. Siddhanta, K. M. Sielewicz, T. Siemiarczuk, D. Silvermyr, C. Silvestre, G. Simatovic, G. Simonetti, R. Singaraju, R. Singh, V. Singhal, T. Sinha, B. Sitar, M. Sitta, T. B. Skaali, M. Slupecki, N. Smirnov, R. J. M. Snellings, T. W. Snellman, J. Song, M. Song, F. Soramel, S. Sorensen, F. Sozzi, E. Spiriti, I. Sputowska, B. K. Srivastava, J. Stachel, I. Stan, P. Stankus, E. Stenlund, J. H. Stiller, D. Stocco, P. Strmen, A. A. P. Suaide, T. Sugitate, C. Suire, M. Suleymanov, M. Suljic, R. Sultanov, M. Šumbera, S. Sumowidagdo, K. Suzuki, S. Swain, A. Szabo, I. Szarka, A. Szczepankiewicz, M. Szymanski, U. Tabassam, J. Takahashi, G. J. Tambave, N. Tanaka, M. Tarhini, M. Tariq, M. G. Tarzila, A. Tauro, G. Tejeda Muñoz, A. Telesca, K. Terasaki, C. Terrevoli, B. Teyssier, D. Thakur, S. Thakur, D. Thomas, R. Tieulent, A. Tikhonov, A. R. Timmins, A. Toia, S. Tripathy, S. Trogolo, G. Trombetta, V. Trubnikov, W. H. Trzaska, B. A. Trzeciak, T. Tsuji, A. Tumkin, R. Turrisi, T. S. Tveter, K. Ullaland, E. N. Umaka, A. Uras, G. L. Usai, A. Utrobicic, M. Vala, J. Van Der Maarel, J. W. Van Hoorne, M. van Leeuwen, T. Vanat, P. Vande Vyvre, D. Varga, A. Vargas, M. Vargyas, R. Varma, M. Vasileiou, A. Vasiliev, A. Vauthier, O. Vázquez Doce, V. Vechernin, A. M. Veen, A. Velure, E. Vercellin, S. Vergara Limón, R. Vernet, R. Vértesi, L. Vickovic, S. Vigolo, J. Viinikainen, Z. Vilakazi, O. Villalobos Baillie, A. Villatoro Tello, A. Vinogradov, L. Vinogradov, T. Virgili, V. Vislavicius, A. Vodopyanov, M. A. Völkl, K. Voloshin, S. A. Voloshin, G. Volpe, B. von Haller, I. Vorobyev, D. Voscek, D. Vranic, J. Vrláková, B. Wagner, J. Wagner, H. Wang, M. Wang, D. Watanabe, Y. Watanabe, M. Weber, S. G. Weber, D. F. Weiser, J. P. Wessels, U. Westerhoff, A. M. Whitehead, J. Wiechula, J. Wikne, G. Wilk, J. Wilkinson, G. A. Willems, M. C. S. Williams, B. Windelband, W. E. Witt, S. Yalcin, P. Yang, S. Yano, Z. Yin, H. Yokoyama, I.-K. Yoo, J. H. Yoon, V. Yurchenko, V. Zaccolo, A. Zaman, C. Zampolli, H. J. C. Zanoli, S. Zaporozhets, N. Zardoshti, A. Zarochentsev, P. Závada, N. Zaviyalov, H. Zbroszczyk, M. Zhalov, H. Zhang, X. Zhang, Y. Zhang, C. Zhang, Z. Zhang, C. Zhao, N. Zhigareva, D. Zhou, Y. Zhou, Z. Zhou, H. Zhu, J. Zhu, X. Zhu, A. Zichichi, A. Zimmermann, M. B. Zimmermann, S. Zimmermann, G. Zinovjev, J. Zmeskal, ALICE Collaboration

**Affiliations:** 1 A.I. Alikhanyan National Science Laboratory (Yerevan Physics Institute) Foundation, Yerevan, Armenia; 2 Benemérita Universidad Autónoma de Puebla, Puebla, Mexico; 3 Bogolyubov Institute for Theoretical Physics, Kiev, Ukraine; 4 Department of Physics and Centre for Astroparticle Physics and Space Science (CAPSS), Bose Institute, Kolkata, India; 5 Budker Institute for Nuclear Physics, Novosibirsk, Russia; 6 California Polytechnic State University, San Luis Obispo, CA USA; 7 Central China Normal University, Wuhan, China; 8 Centre de Calcul de l’IN2P3, Villeurbanne, Lyon, France; 9 Centro de Aplicaciones Tecnológicas y Desarrollo Nuclear (CEADEN), Havana, Cuba; 10 Centro de Investigaciones Energéticas Medioambientales y Tecnológicas (CIEMAT), Madrid, Spain; 11 Centro de Investigación y de Estudios Avanzados (CINVESTAV), Mexico City, Mérida, Mexico; 12 Centro Fermi-Museo Storico della Fisica e Centro Studi e Ricerche “Enrico Fermi’, Rome, Italy; 13 Chicago State University, Chicago, IL USA; 14 China Institute of Atomic Energy, Beijing, China; 15 COMSATS Institute of Information Technology (CIIT), Islamabad, Pakistan; 16 Departamento de Física de Partículas and IGFAE, Universidad de Santiago de Compostela, Santiago de Compostela, Spain; 17 Department of Physics, Aligarh Muslim University, Aligarh, India; 18 Department of Physics, Ohio State University, Columbus, OH USA; 19 Department of Physics, Sejong University, Seoul, South Korea; 20 Department of Physics, University of Oslo, Oslo, Norway; 21 Department of Physics and Technology, University of Bergen, Bergen, Norway; 22 Dipartimento di Fisica dell’Università ‘La Sapienza’ and Sezione INFN, Rome, Italy; 23 Dipartimento di Fisica dell’Università and Sezione INFN, Cagliari, Italy; 24 Dipartimento di Fisica dell’Università and Sezione INFN, Trieste, Italy; 25 Dipartimento di Fisica dell’Università and Sezione INFN, Turin, Italy; 26 Dipartimento di Fisica e Astronomia dell’Università and Sezione INFN, Bologna, Italy; 27 Dipartimento di Fisica e Astronomia dell’Università and Sezione INFN, Catania, Italy; 28 Dipartimento di Fisica e Astronomia dell’Università and Sezione INFN, Padua, Italy; 29 Dipartimento di Fisica ‘E.R. Caianiello’ dell’Università and Gruppo Collegato INFN, Salerno, Italy; 30 Dipartimento DISAT del Politecnico and Sezione INFN, Turin, Italy; 31 Dipartimento di Scienze e Innovazione Tecnologica dell’Università del Piemonte Orientale and INFN Sezione di Torino, Alessandria, Italy; 32 Dipartimento Interateneo di Fisica ‘M. Merlin’ and Sezione INFN, Bari, Italy; 33 Division of Experimental High Energy Physics, University of Lund, Lund, Sweden; 34 European Organization for Nuclear Research (CERN), Geneva, Switzerland; 35 Excellence Cluster Universe, Technische Universität München, Munich, Germany; 36 Faculty of Engineering, Bergen University College, Bergen, Norway; 37 Faculty of Mathematics, Physics and Informatics, Comenius University, Bratislava, Slovakia; 38 Faculty of Nuclear Sciences and Physical Engineering, Czech Technical University in Prague, Prague, Czech Republic; 39 Faculty of Science, P.J. Šafárik University, Košice, Slovakia; 40 Faculty of Technology, Buskerud and Vestfold University College, Tonsberg, Norway; 41 Frankfurt Institute for Advanced Studies, Johann Wolfgang Goethe-Universität Frankfurt, Frankfurt, Germany; 42 Gangneung-Wonju National University, Gangneung, South Korea; 43 Department of Physics, Gauhati University, Guwahati, India; 44 Helmholtz-Institut für Strahlen- und Kernphysik, Rheinische Friedrich-Wilhelms-Universität Bonn, Bonn, Germany; 45 Helsinki Institute of Physics (HIP), Helsinki, Finland; 46 Hiroshima University, Hiroshima, Japan; 47 Indian Institute of Technology Bombay (IIT), Mumbai, India; 48 Indian Institute of Technology Indore, Indore, India; 49 Indonesian Institute of Sciences, Jakarta, Indonesia; 50 Inha University, Incheon, South Korea; 51 Institut de Physique Nucléaire d’Orsay (IPNO), Université Paris-Sud, CNRS-IN2P3, Orsay, France; 52 Institute for Nuclear Research, Academy of Sciences, Moscow, Russia; 53 Institute for Subatomic Physics of Utrecht University, Utrecht, Netherlands; 54 Institute for Theoretical and Experimental Physics, Moscow, Russia; 55 Institute of Experimental Physics, Slovak Academy of Sciences, Košice, Slovakia; 56 Institute of Physics, Academy of Sciences of the Czech Republic, Prague, Czech Republic; 57 Institute of Physics, Bhubaneswar, India; 58 Institute of Space Science (ISS), Bucharest, Romania; 59 Institut für Informatik, Johann Wolfgang Goethe-Universität Frankfurt, Frankfurt, Germany; 60 Institut für Kernphysik, Johann Wolfgang Goethe-Universität Frankfurt, Frankfurt, Germany; 61 Institut für Kernphysik, Westfälische Wilhelms-Universität Münster, Münster, Germany; 62 Instituto de Ciencias Nucleares, Universidad Nacional Autónoma de México, Mexico City, Mexico; 63 Instituto de Física, Universidade Federal do Rio Grande do Sul (UFRGS), Porto Alegre, Brazil; 64 Instituto de Física, Universidad Nacional Autónoma de México, Mexico City, Mexico; 65 IRFU, CEA, Université Paris-Saclay, F-91191 Gif-sur-Yvette France, Saclay, France; 66 iThemba LABS, National Research Foundation, Somerset West, South Africa; 67 Joint Institute for Nuclear Research (JINR), Dubna, Russia; 68 Konkuk University, Seoul, South Korea; 69 Korea Institute of Science and Technology Information, Daejeon, South Korea; 70 KTO Karatay University, Konya, Turkey; 71 Laboratoire de Physique Corpusculaire (LPC), Clermont Université, Université Blaise Pascal, CNRS-IN2P3, Clermont-Ferrand, France; 72 Laboratoire de Physique Subatomique et de Cosmologie, Université Grenoble-Alpes, CNRS-IN2P3, Grenoble, France; 73 Laboratori Nazionali di Frascati, INFN, Frascati, Italy; 74 Laboratori Nazionali di Legnaro, INFN, Legnaro, Italy; 75 Lawrence Berkeley National Laboratory, Berkeley, CA USA; 76 Moscow Engineering Physics Institute, Moscow, Russia; 77 Nagasaki Institute of Applied Science, Nagasaki, Japan; 78 Physics Department, National and Kapodistrian University of Athens, Athens, Greece; 79 National Centre for Nuclear Studies, Warsaw, Poland; 80 National Institute for Physics and Nuclear Engineering, Bucharest, Romania; 81 National Institute of Science Education and Research, Bhubaneswar, India; 82 National Nuclear Research Center, Baku, Azerbaijan; 83 National Research Centre Kurchatov Institute, Moscow, Russia; 84 Niels Bohr Institute, University of Copenhagen, Copenhagen, Denmark; 85 Nikhef, Nationaal instituut voor subatomaire fysica, Amsterdam, Netherlands; 86 Nuclear Physics Group, STFC Daresbury Laboratory, Daresbury, UK; 87 Nuclear Physics Institute, Academy of Sciences of the Czech Republic, Řež u Prahy, Czech Republic; 88 Oak Ridge National Laboratory, Oak Ridge, TN USA; 89 Petersburg Nuclear Physics Institute, Gatchina, Russia; 90 Physics Department, Creighton University, Omaha, NE USA; 91 Physics Department, Panjab University, Chandigarh, India; 92 Physics Department, University of Cape Town, Cape Town, South Africa; 93 Physics Department, University of Jammu, Jammu, India; 94 Physics Department, University of Rajasthan, Jaipur, India; 95 Physikalisches Institut, Eberhard Karls Universit?t Tübingen, Tübingen, Germany; 96 Physikalisches Institut, Ruprecht-Karls-Universität Heidelberg, Heidelberg, Germany; 97 Physik Department, Technische Universität München, Munich, Germany; 98 Purdue University, West Lafayette, IN USA; 99 Pusan National University, Pusan, South Korea; 100 Research Division and ExtreMe Matter Institute EMMI, GSI Helmholtzzentrum für Schwerionenforschung GmbH, Darmstadt, Germany; 101 Rudjer Bošković Institute, Zagreb, Croatia; 102 Russian Federal Nuclear Center (VNIIEF), Sarov, Russia; 103 Saha Institute of Nuclear Physics, Kolkata, India; 104 School of Physics and Astronomy, University of Birmingham, Birmingham, UK; 105 Sección Física, Departamento de Ciencias, Pontificia Universidad Católica del Perú, Lima, Peru; 106 Sezione INFN, Bari, Italy; 107 Sezione INFN, Bologna, Italy; 108 Sezione INFN, Cagliari, Italy; 109 Sezione INFN, Catania, Italy; 110 Sezione INFN, Padua, Italy; 111 Sezione INFN, Rome, Italy; 112 Sezione INFN, Trieste, Italy; 113 Sezione INFN, Turin, Italy; 114 SSC IHEP of NRC Kurchatov institute, Protvino, Russia; 115 Stefan Meyer Institut für Subatomare Physik (SMI), Vienna, Austria; 116 SUBATECH, Ecole des Mines de Nantes, Université de Nantes, CNRS-IN2P3, Nantes, France; 117 Suranaree University of Technology, Nakhon Ratchasima, Thailand; 118 Technical University of Košice, Košice, Slovakia; 119 Technical University of Split FESB, Split, Croatia; 120 The Henryk Niewodniczanski Institute of Nuclear Physics, Polish Academy of Sciences, Kraków, Poland; 121 Physics Department, The University of Texas at Austin, Austin, TX USA; 122 Universidad Autónoma de Sinaloa, Culiacán, Mexico; 123 Universidade de São Paulo (USP), São Paulo, Brazil; 124 Universidade Estadual de Campinas (UNICAMP), Campinas, Brazil; 125 Universidade Federal do ABC, Santo Andre, Brazil; 126 University of Houston, Houston, TX USA; 127 University of Jyväskylä, Jyväskylä, Finland; 128 University of Liverpool, Liverpool, UK; 129 University of Tennessee, Knoxville, TN USA; 130 University of the Witwatersrand, Johannesburg, South Africa; 131 University of Tokyo, Tokyo, Japan; 132 University of Tsukuba, Tsukuba, Japan; 133 University of Zagreb, Zagreb, Croatia; 134 Université de Lyon, Université Lyon 1, CNRS/IN2P3, IPN-Lyon, Villeurbanne, Lyon, France; 135 Université de Strasbourg, CNRS, IPHC UMR 7178, 67000 Strasbourg, France; 136 Università degli Studi di Pavia, Pavia, Italy; 137 Università di Brescia, Brescia, Italy; 138 V. Fock Institute for Physics, St. Petersburg State University, St. Petersburg, Russia; 139 Variable Energy Cyclotron Centre, Kolkata, India; 140 Warsaw University of Technology, Warsaw, Poland; 141 Wayne State University, Detroit, MI USA; 142 Wigner Research Centre for Physics, Hungarian Academy of Sciences, Budapest, Hungary; 143 Yale University, New Haven, CT USA; 144 Yonsei University, Seoul, South Korea; 145 Zentrum für Technologietransfer und Telekommunikation (ZTT), Fachhochschule Worms, Worms, Germany; 146 CERN, 1211 Geneva 23, Switzerland

## Abstract

The transverse momentum distributions of the strange and double-strange hyperon resonances ($\Sigma (1385)^{\pm }$, $\Xi (1530)^{0}$) produced in p–Pb collisions at $\sqrt{s_{\mathrm{NN}}}= 5.02$ TeV were measured in the rapidity range $-0.5< y_\mathrm {CMS}<0$ for event classes corresponding to different charged-particle multiplicity densities, $\langle$d$N_{\mathrm{ch}}$/d$\eta _{\mathrm{lab}}\rangle$. The mean transverse momentum values are presented as a function of $\langle$d$N_{\mathrm{ch}}$/d$\eta _{\mathrm{lab}}\rangle$, as well as a function of the particle masses and compared with previous results on hyperon production. The integrated yield ratios of excited to ground-state hyperons are constant as a function of $\langle$d$N_{\mathrm{ch}}$/d$\eta _{\mathrm{lab}}\rangle$. The equivalent ratios to pions exhibit an increase with $\langle$d$N_{\mathrm{ch}}$/d$\eta _{\mathrm{lab}}\rangle$, depending on their strangeness content.

## Introduction

Hadrons containing one or more strange quarks have been studied extensively over past decades in connection with the study of quark-gluon plasma [1, 2]. Enhanced hyperon yields were observed in heavy-ion collisions with respect to those measured in proton-proton (pp) collisions at the same centre-of-mass energy [3–6]. These enhancements were found to be consistent with those expected from thermal statistical model calculations using a grand canonical ensemble [7]. The canonical [8, 9] approach is suggested to explain the relatively suppressed multi-strange baryon yields in smaller collision systems such as $\mathrm{pp}$, proton-nucleus (p–Pb) and peripheral heavy-ion collisions [10].

Short-lived resonances, such as $\mathrm{K}^{*0}$ and ${\Sigma (1385)}^{\pm }$, can be used in heavy-ion collisions to study the hadronic medium between chemical and kinetic freeze-out [11]. Chemical and kinetic freeze-out define the points in time, respectively, when hadron abundances and the momenta of particles stop changing. Decay products of resonances are subject to re-scattering processes and emerge after kinetic decoupling with little memory of the source. Regeneration processes, conversely, increase the resonance yield [12]. If re-scattering processes are dominant over regeneration processes, the measured yield of resonances is expected to be reduced. Moreover, the longer the time between chemical and kinetic freeze-out, the greater the expected reduction.

Recently, the ALICE collaboration reported results on $\, \mathrm{K}^{*0}$, $\phi$, $\Xi ^-$ and $\Omega ^-$ in $\mathrm{pp}$  and $\text{ p--Pb }$  collisions [10, 13, 14] in addition to $\text{ Pb--Pb }$ data [6, 15]. The evolution of the mean transverse momenta (${\langle p_{\mathrm{T}}\rangle }$) of mesons and multi-strange baryons were presented as a function of charged-particle multiplicity and particle mass. The observed decrease of the resonance to ground-state ratio $\,{\mathrm{K}}^{{*0}}/{\mathrm{K}}^{{-}}$ has been suggested as an indication of re-scattering processes in the hadronic medium, as first observed in Pb–Pb collisions [15].

This paper reports on the hyperon resonances ${\Sigma (1385)}^{{\pm }}$ ($c\tau = 5.48$ fm, *uus* or *dds* [16]) and ${\Xi (1530)}^{{0}}$ ($c\tau = 22$ fm, *uss* [16]), measured in p–Pb collisions at $\sqrt{s_{\mathrm{NN}}}= 5.02$ TeV. The corresponding results for pp collisions have been previously published in [17]. The results presented in this paper complement the p–Pb results given in [10, 14]. The measured $p_{\mathrm{T}}$ spectra, yields and mean transverse momenta are presented for different multiplicity classes. Yield ratios of excited to ground-state hyperons are studied as a function of event multiplicity and compared with model predictions [7, 18–20]. Considering the similar lifetimes of ${\Sigma (1385)}^{\pm }$ and $\mathrm{K}^{*0}$, a decrease of the ${\Sigma (1385)}^{\pm }/\Lambda$ ratio, consistent with the decrease observed for the $\mathrm{K}^{*0}/\mathrm{K}^{-}$ ratio, is expected for increasing system sizes. Hyperon to pion ratios are also presented and compared to the results for ground-state hyperons with the same strangeness contents.

In this paper, the short notations ${\Sigma ^{*{\pm }}}$ and ${\Xi }^{*0}$ are adopted for ${\Sigma (1385)}^{\pm }$ and ${\Xi (1530)}^{0}$. Moreover, the notations ${\Sigma }^{*\pm }$ and $\Xi ^{*0}$ include the respective anti-particles, namely ${\Sigma }^{*\pm }$ includes ${\Sigma }^{*+}$, ${\Sigma }^{*-}$, and their anti-particles, while $\Xi ^{*0}$ means $\Xi ^{*0}$ and $\overline{\Xi ^*}^0$, unless otherwise indicated.

## Experimental setup and event selection

A description of the ALICE detector and of its performance during the LHC Run 1 (2010–2013) can be found in [21, 22]. The data sample analysed in this paper was recorded during the LHC p–Pb run at $\sqrt{s_{\mathrm{NN}}}$ = 5.02 TeV in 2013. Due to the asymmetric energies of the proton (4 TeV) and lead ion (1.57 A TeV) beams, the centre-of-mass system in the nucleon-nucleon frame is shifted in rapidity by $\Delta y_{\mathrm {NN}}$ = 0.465 towards the direction of the proton beam with respect to the laboratory frame of the ALICE detector [14]. For the analysed p–Pb data set, the direction of the proton beam was towards the ALICE muon spectrometer, the so-called “C” side, standing for negative rapidities; conversely, the Pb beam circulated towards positive rapidities, labelled as “A” side in the following. The analysis in this paper was carried out at midrapidity, in the rapidity window $-0.5 < y_{\mathrm {CMS}}<$ 0.

The minimum-bias trigger during the p–Pb run was configured to select events by requiring a logical OR of signals in V0A and V0C [22], two arrays of 32 scintillator detectors covering the full azimuthal angle in the pseudorapidity regions 2.8 $< \eta _{\mathrm {lab}}<$ 5.1 and $-3.7< \eta _{\mathrm {lab}} < -1.7$, respectively [23]. In the data analysis it was required to have a coincidence of signals in both V0A and V0C in order to reduce the contamination from single-diffractive and electromagnetic interactions. This left only non-single diffractive (NSD) events, which amount for a total of 100 million events, in the minimum-bias (MB) sample corresponding to an integrated luminosity of about 50 $\upmu$b$^{-1}$.

The combined V0A and V0C information discriminates beam-beam interactions from background collisions in the interaction region. Further background suppression was applied in the offline analysis using time information from two neutron zero degree calorimeters (ZDC) [22], as in previous p–Pb analyses [24]. Pile-up events due to more than one collision in the region of beam interaction were excluded by using the silicon pixel detector (SPD) in the inner tracking system (ITS) [22]. The primary vertex (PV) is determined by tracks reconstructed in the ITS and time projection chamber (TPC), and track segments in the SPD [22, 23]. MB events are selected when the PV is positioned along the beam axis within ${\pm }$10 cm from the centre of the ALICE detector.

The MB events were divided into several multiplicity classes according to the accumulated charge in the forward V0A detector [25]. The ${\Sigma }^{*{\pm }}$ resonances are reconstructed in the multiplicity classes 0–20, 20–60, and 60–100%, whereas the $\Xi ^{*0}$ analysis is carried out in four classes, namely 0–20, 20–40, 40–60 and 60–100%. To each multiplicity class corresponds a mean charged-particle multiplicity ($\langle$d$N_{\mathrm{ch}}$/d$\eta _{\mathrm{lab}}\rangle$), measured at midrapidity ($|\eta _{\mathrm {lab}}|< 0.5$), as shown in Table 1.

**Table 1:** Mean charged-particle multiplicity densities ($\langle$d$N_{\mathrm{ch}}$/d$\eta _{\mathrm{lab}}\rangle$) measured at midrapidity ($|\eta _{\mathrm {lab}}|< 0.5$) [23], corresponding to the multiplicity classes defined using the V0A detector [25] in $\text{ p--Pb }$ collisions at $\sqrt{s_{\mathrm{NN}}}$ = 5.02 TeV

V0A percentile (%)	$\langle$d$N_{\mathrm{ch}}$/d$\eta _{\mathrm{lab}}\rangle$
0–20	35.6 ± 0.8
20–40	23.2 ± 0.5
20–60	19.7 ± 0.5
40–60	16.1 ± 0.4
60–100	7.1 ± 0.2
0–100	17.4 ± 0.7

## Data analysis

### Track and topological selections

Table 2 summarizes the relevant information on the measured hyperon resonances, namely the decay modes used in this analysis and their branching ratios. In the case of $\Sigma ^{*\pm }$, all states $\Sigma ^{*+}$, $\Sigma ^{*-}$, $\overline{\Sigma }^{*-}$ and $\overline{\Sigma }^{*+}$ were separately analysed, while the $\Xi ^{*0}$ analysis always includes the charge-conjugated anti-particle, $\overline{\Xi }^{*0}$ due to the limited statistics of the dataset.

**Table 2:** Properties of the measured resonances and decay modes used in this analysis with total branching ratios [16], obtained as the products of respective branching ratios of daughter particles

	Mass (MeV/c$^{2}$)	Width (MeV/c$^{2}$)	Decay modes used	Total B.R. (%)
$\Sigma$(1385)$^{+}$	1382.80 ± 0.35	36.0 ± 0.7	$\Lambda \pi ^{+}\rightarrow (p\pi ^-)\pi ^+$	55.6 ± 1.1
$\Sigma$(1385)$^{-}$	1387.2 ± 0.5	39.4 ± 2.1	$\Lambda \pi ^{-}\rightarrow (p\pi ^-)\pi ^-$	
$\Xi$(1530)$^{0}$	1531.80 ± 0.32	9.1 ± 0.5	$\Xi ^{-} \pi ^{+}\rightarrow (\Lambda \pi ^-)\pi ^+\rightarrow ((p\pi ^-)\pi ^-)\pi ^+$	42.6 ± 0.3

In comparison with the $\Sigma ^{*\pm }$ and $\Xi ^{*0}$ analysis carried out in pp collisions at $\sqrt{s}$ = 7 TeV [17], track and topological selections were revised and adapted to the p–Pb dataset; this is notably the case for $\Xi ^{*0}$. Pions from strong decays of both $\Sigma ^{*\pm }$ and $\Xi ^{*0}$ were selected according to the criteria for primary tracks. As summarized in Table 3, all charged tracks were selected with $p_{\mathrm{T}}$ > 0.15  $\mathrm{GeV/c}$  and $|\eta _{\mathrm {lab}}| <0.8$, as described in Ref. [22]. The primary tracks were chosen with the distance of closest approach (DCA) to PV of less than 2 cm along the longitudinal direction (DCA$_z$) and lower than 7$\sigma _r$ in the transverse plane (DCA$_r$), where $\sigma _r$ is the resolution of DCA$_r$. The $\sigma _r$ is strongly $p_{\mathrm{T}}$-dependent and lower than 100 $\upmu$m for $p_{\mathrm{T}}>$ 0.5 $\mathrm{GeV/c}$ [22]. To ensure a good track reconstruction quality, candidate tracks were required to have at least one hit in one of the two innermost layers (SPD) of the ITS and to have at least 70 reconstructed points in the TPC, out of a maximum of 159. The particle identification (PID) criteria for all decay daughters are based on the requirement that the specific energy loss (d*E*/d*x*) is measured in the TPC within three standard deviations ($\sigma _\mathrm {TPC}$) from the expected value (d*E*/d$x_\mathrm{{exp}}$), computed using a Bethe–Bloch parametrization [22].

**Table 3:** Track selections common to all decay daughters and primary track selections applied to the charged pions from decays of $\Sigma ^{*\pm }$ and $\Xi ^{*0}$

Common track selections	$|\eta _{\mathrm {lab}}|$	<0.8
	$p_{\mathrm{T}}$	>0.15 GeV/c
	PID |(d*E* / d$$x)-$$(d*E* / d$$x)_\mathrm{{exp}}|$$	<3 $$\sigma _\mathrm{{TPC}}$$
Primary track selections	DCA$_z$ to PV	<2 cm
	DCA$_r$ to PV	<7 $\sigma _r$ ($p_\mathrm {T}$)
	number of SPD points	$\ge$1
	number of TPC points	>70

Since pions and protons from weak decay of $\Lambda$ ($\textit{c}\tau = 7.89$ cm [16]) and pions from weak decay of $\Xi ^{-}$ ($\textit{c}\tau = 4.91$ cm [16]) are produced away from the PV, specific topological and track selection criteria, as summarized in Table 4, were applied [10, 17, 26].

**Table 4:** Topological and track selection criteria

	$\Sigma ^{*\pm }$	$\Xi ^{*0}$
DCA$_r$ of $\Lambda$ decay products to PV	>0.05 cm	>0.06 cm
DCA between $\Lambda$ decay products	<1.6 cm	<1.4 cm
DCA of $\Lambda$ to PV	<0.3 cm	>0.015 cm
cos$\theta _\Lambda$	>0.99	>0.875
$r(\Lambda )$	1.4 $<r(\Lambda )<$ 100 cm	0.2 $<r(\Lambda )<$ 100 cm
$|M_{p\pi } - m_\Lambda |$	<10 MeV/c$^{2}$	<7 MeV/c$^{2}$
DCA$_r$ of pion (from $\Xi ^{-}$) to PV		>0.015 cm
DCA between $\Xi ^{-}$ decay products		<1.9 cm
cos$\theta _\Xi$		>0.981
$r(\Xi ^-)$		0.2 $<r(\Xi ^-)<$ 100 cm
$|M_{\Lambda \pi } - m_\Xi |$		<7 MeV/c$^{2}$

**Fig. 1:**
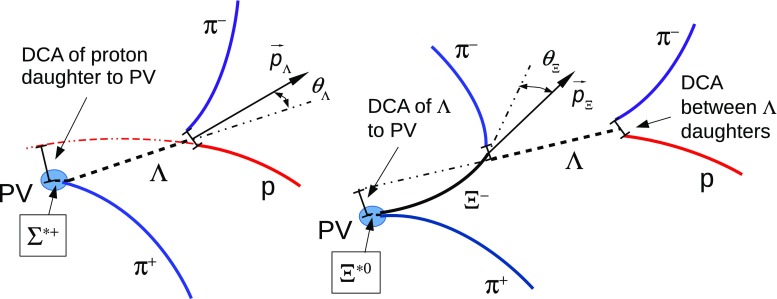
Sketch of the decay modes for $\Sigma ^{*+}$ (*left*) and $\Xi ^{*0}$ (*right*) and depiction of the track and topological selection criteria

In the analysis of $\Sigma ^{*\pm }$, secondary $\pi$ and p from $\Lambda$ decays were selected with a DCA between the two tracks of less than 1.6 cm and with a DCA$_r$ to the PV greater than 0.05 cm, to remove most primary tracks. For $\Sigma ^{*-}$ and $\overline{\Sigma }^{*+}$, the DCA of $\Lambda$ to the PV must be smaller than 0.3 cm in order to remove most of the primary weakly-decaying $\Xi (1321)^{-}$ and $\overline{\Xi }(1321)^{+}$, which share the same decay channel. The $\Lambda$ invariant mass ($M_{p\pi }$) was selected within ± 10 MeV/c$^{2}$ of the particle data group (PDG) value ($m_\Lambda =1115.683\pm 0.006$ MeV/c$^{2}$) [16], the cosine of the pointing angle $\theta _\Lambda$ (the angle between the sum of daughter momenta and the line that connects the PV and the decay vertex, as shown in Fig. 1) was requested to be greater than 0.99, and the radius of the fiducial volume $r(\Lambda )$ (the distance between the PV and the decay vertex) was requested to be between 1.4 and 100 cm.

In the analysis of $\Xi ^{*0}$, $\Lambda$ and $\pi$ from $\Xi ^{-}$ were selected with a DCA of less than 1.9 cm and with a DCA$_r$ to the PV greater than 0.015 cm. The $\Lambda$ daughter particles ($\pi$ and p) were required to have a DCA$_r$ to the PV greater than 0.06 cm, while the DCA between the two particles was required to be less than 1.4 cm. Cuts on the invariant mass, the cosine of the pointing angle ($\theta _\Lambda$, $\theta _\Xi$) and the radius of the fiducial volume ($r(\Lambda )$, $r(\Xi )$) in Table 4 were applied to optimize the balance of purity and efficiency of each particle sample.

### Signal extraction

**Fig. 2:**
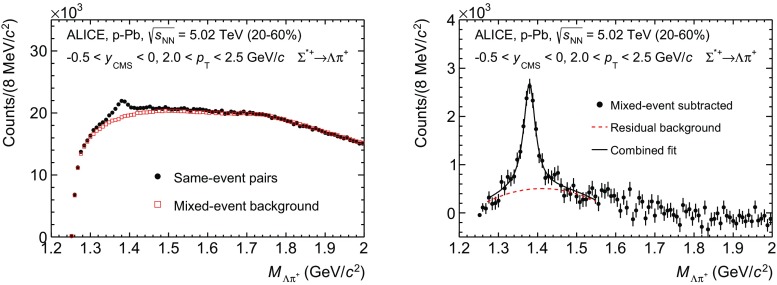
(*Left*) the $\Lambda \pi ^{+}$ invariant mass distribution (same-event pairs) in 2.0 < $p_{\mathrm{T}}$ < 2.5 $\mathrm{GeV/c}$ and for the multiplicity class 20–60%. The background shape, using pairs from different events (mixed-event background), is normalised to the counts in 1.9 $<M_{\Lambda \pi }<$ 2.0 $\mathrm{GeV/c}^{2}$. (*Right*) the invariant mass distribution after subtraction of the mixed-event background. The *solid curve* represents the combined fit, while the *dashed line* describes the residual background

**Fig. 3:**
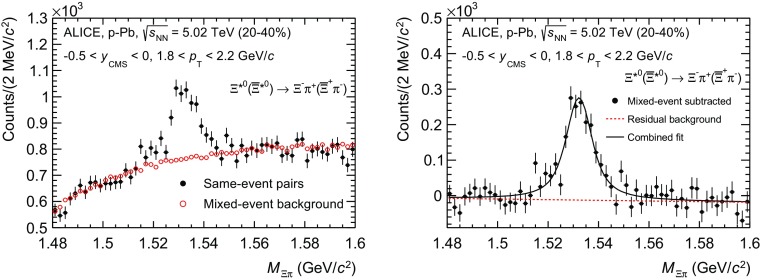
(*Left*) the $\Xi ^{\mp }\pi ^{\pm }$ invariant mass distribution (same-event pairs) in 1.8 < $p_{\mathrm{T}}$ < 2.2 $\mathrm{GeV/c}$ and for the multiplicity class 20–40%. The background shape, using pairs from different events (mixed-event background), is normalised to the counts in $1.49< M_{\Xi \pi } < 1.51$ $\mathrm{GeV/c}^{2}$ and $1.56< M_{\Xi \pi } < 1.58$ $\mathrm{GeV/c}^{2}$. (*Right*) the invariant mass distribution after subtraction of the mixed-event background. The *solid curve* represents the combined fit, while the *dashed line* describes the residual background

The $\Sigma ^{*\pm }$ and $\Xi ^{*0}$ signals were reconstructed by invariant-mass analysis of candidates for the decay products in each transverse momentum interval of the resonance particle, and for each multiplicity class. Examples of invariant-mass distributions are presented in the left panels of Figs. 2 and 3 for $\Sigma ^{*+}\rightarrow \Lambda \pi ^+$ and $\Xi ^{*0}$($\overline{\Xi }^{*0}$) $\rightarrow$ $\Xi ^-\pi ^+$($\Xi ^+\pi ^-$), respectively.^1^

Since the resonance decay products originate from a position which is indistinguishable from the PV, a significant combinatorial background is present. These background distributions were determined by means of a mixed-event technique, by combining uncorrelated decay products from 5 and 20 different events in the $\Sigma ^{*\pm }$ and $\Xi ^{*0}$ analyses, respectively. In order to minimise distortions due to different acceptances and to ensure a similar event structure, only tracks from events with similar vertex positions *z* ($|\Delta z|<$ 1 cm) and track multiplicities *n* ($|\Delta n|<$ 10) were taken.

For $\Sigma ^{*\pm }$, the mixed-event background distributions were normalised to a $p_{\mathrm{T}}$-dependent invariant mass region where the mixed-event background and the invariant mass distribution have similar slopes, as shown in Fig. 2 (left). These $p_{\mathrm{T}}$-dependent invariant mass regions range from $1.5~<~M_{\Lambda \pi }<~2.0$ $\mathrm{GeV/c}^{2}$, for the lowest $p_{\mathrm{T}}$ bin, to $1.95<~M_{\Lambda \pi }~<~2.0$ $\mathrm{GeV/c}^{2}$, for the highest $p_{\mathrm{T}}$ bin. More details on the normalisation procedure are provided in Ref. [17]. The contribution of the normalisation to the systematic uncertainty was estimated by selecting different normalisation regions and accounts for less than 1%.

For $\Xi ^{*0}$, the mixed-event background distributions were normalised to two fixed regions, 1.49 < $M_{\Xi \pi }$ < 1.51 $\mathrm{GeV/c}^{2}$ and 1.56< $M_{\Xi \pi }$ < 1.58 $\mathrm{GeV/c}^{2}$,  around the $\Xi ^{*0}$ mass peak (Fig. 3 (left)). These regions were used for all $p_{\mathrm{T}}$ intervals and multiplicity classes, because the background shape is reasonably well reproduced in these regions and the invariant-mass resolution of the reconstructed peaks appears stable, independently of $p_{\mathrm{T}}$. The uncertainty on the normalisation was estimated by varying the normalisation regions and is included in the quoted systematic uncertainty for the signal extraction (Table 5).

For $\Sigma ^{*\pm }$, a combined fit of a second-order polynomial for the residual background description and a Breit–Wigner function with a width fixed to the PDG values [16] for the signal were used in the invariant-mass range of $1.28<M_{\Lambda \pi }<1.55$ $\mathrm{GeV/c}^{2}$. The detector resolution ($\sim$1 MeV/c$^{2}$) is much lower than the $\Sigma ^{*\pm }$ width and was therefore neglected. In the right panel of Fig. 2, the solid and dashed lines show the result of the combined fit and the residual background, respectively. Alternative fit ranges were taken into account in the estimation of the systematic uncertainty. A linear and a cubic parametrization for the residual background were used to study the systematic uncertainty related to the signal extraction.

For $\Xi ^{*0}$, a combined fit of a first-order polynomial for the residual background and a Voigtian function (a convolution of a Breit–Wigner and a Gaussian function accounting for the detector resolution) for the signal was used, as described in Ref. [17].

The raw yields $N^\mathrm{RAW}$ were obtained by integrating the signal function from the combined fit. For $\Sigma ^{*\pm }$, the integration of the Breit–Wigner function was carried out in the invariant mass range between 1.28 and 1.56 $\mathrm{GeV/c}^{2}$. For $\Xi ^{*0}$, the integration of the Voigtian function was done in the mass region between 1.48 and 1.59 $\mathrm{GeV/c}^{2}$. In both cases, corrections for the tails outside the integration region were applied. The statistical uncertainties on the raw yields range between 5 and 15% for $\Sigma ^{*\pm }$ and 2–6% for $\Xi ^{*0}$, respectively.

### Corrections and normalisation

**Fig. 4:**
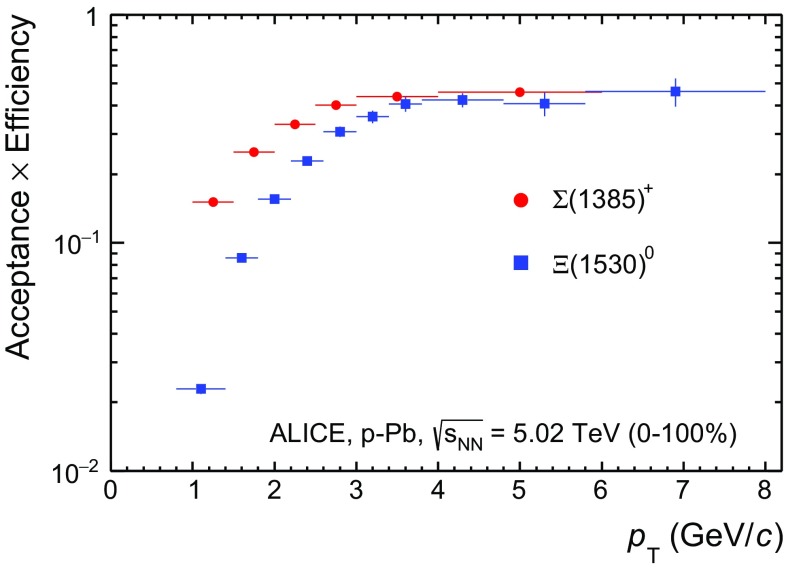
The geometrical acceptance and the reconstruction efficiency (A $\times$ $\varepsilon$) for $\Sigma ^{*+}$ and $\Xi ^{*0}$ in $-0.5 < y_{\mathrm {CMS}^{\mathrm {MC}}}<$ 0 for minimum-bias events, obtained with DPMJET 3.05 [19] and GEANT 3.1  [27]. Only statistical uncertainties are shown

The raw yields were corrected for the geometrical acceptance and the reconstruction efficiency (A $\times$ $\varepsilon$) of the detector (Fig. 4) and by branching ratios (total B.R. in Table 2). By using the DPMJET 3.05 event generator [19] and the GEANT 3.21 package [27], a sample of about 100 million p–Pb events was simulated and reconstructed in order to compute the corrections. The distributions of $A\times \varepsilon$ were obtained from the ratio between the number of reconstructed hyperons ($\Sigma ^{*\pm }$ or $\Xi ^{*0}$) and the number of generated hyperons in the same $p_{\mathrm{T}}$ and rapidity interval. Inefficiencies in the vertex reconstruction have a negligible effect for all multiplicity classes except 60–100%, where a correction factor of 1.03 has to be applied to the raw yields.

The product $A\times \epsilon$ for MB events is shown in Fig. 4 for $\Sigma ^{*+}$ and $\Xi ^{*0}$. Since the correction factors for different multiplicity classes are in agreement with those from MB events within statistical uncertainty, the latter were used for all multiplicity classes. For $\Sigma ^{*+}$ and $\Sigma ^{*-}$, the correction factors were the same. In the case of $\overline{\Sigma }^{*+}$ and $\overline{\Sigma }^{*-}$, correction factors were around 10% higher at low $p_{\mathrm{T}}$, as expected due to the different interaction cross sections of proton and antiprotons in the detector’s material  [28].

Finally, the yields were normalised to the number of events analysed in each multiplicity class, as defined in Table 1. The MB spectra were instead normalised to the number of NSD events after applying the correction factors for trigger efficiency and event selection, primary vertex reconstruction and selection, resulting in a total scaling factor of 0.964 [14].

### Systematic uncertainties

Systematic effects due to the global tracking efficiency, track and topological selection cuts, PID, mass window selection ($\Xi ^\pm$), vertex selection, signal extraction and uncertainties on the knowledge of the material budget and branching ratio were studied for each $p_{\mathrm{T}}$ interval and multiplicity class by comparing different choices of selection criteria. The results are summarized in Table 5.

**Table 5:** Summary of the systematic uncertainties on the differential yield, $\mathrm{d}^2N/(\mathrm{d}p_\mathrm{T}\mathrm{d}y)$. Minimum and maximum values in all $p_{\mathrm{T}}$ intervals and multiplicity classes are shown for each source

Source of uncertainty	$\Sigma ^{*\pm }$ (%)	$\Xi ^{*0}$ (%)
$p_{\mathrm{T}}$-dependent		
Tracking efficiency	3	3
Tracks selection	1–2	1–2
Topological selection	1–4	1–2
PID	1–3	3–7
Signal extraction	2–5	1–5
Mass window ($\Xi ^\pm$)	–	4
Vertex selection	1–2	3
$p_{\mathrm{T}}$-independent		
Material budget	4	4
Branching ratio	1.1	0.3
Total	7–9	8–12

Each source of systematic effects was first requested to pass a consistency check, testing whether a change in selection criteria prevents statistically significant differences in the reconstructed yields [29]. If the source failed the consistency check, the deviation between the default yield and the alternative one obtained by varying the selection was taken as systematic uncertainty. Sources which did not provide statistically significant differences are not listed in Table 5 (e.g. $\Lambda$ invariant mass window). The uncertainty for the $\Sigma ^{*\pm }$ yield is taken as the average of the uncertainties for $\Sigma ^{*+}$, $\overline{\Sigma }^{*-}$, $\Sigma ^{*-}$, and $\overline{\Sigma }^{*+}$.

For $\Sigma ^{*\pm }$, the main contribution to the total systematic uncertainty originates from the signal extraction, while for $\Xi ^{*0}$ the main contribution is from the PID. The signal extraction includes variations of the background normalisation region, choice of the integration interval of the raw yield determination and, in the case of $\Sigma ^{*\pm }$, order of the polynomial for describing the residual background. Also, an alternative method, which integrates the signal distribution by summing the bin contents, provides negligible differences.

Table 5 reports the minimum and maximum of the systematic uncertainty from each source. The systematic uncertainty in each $p_{\mathrm{T}}$ interval is obtained as the quadratic sum of all contributions, except the $p_{\mathrm{T}}$-independent uncertainties, which affect only the normalisation (see Sect. 4.1). The uncertainties which are dependent on multiplicity and uncorrelated across different multiplicity bins were treated separately. Topological selections, signal extraction and PID give the dominant contributions to the uncertainties uncorrelated across multiplicity. These uncertainties were estimated to be within 3% (5%), which represents a fraction of 35% (50%) of the total systematic uncertainty for $\Sigma ^{*\pm }$ ($\Xi ^{*0}$).

## Results and discussion

### Transverse momentum spectra

**Fig. 5:**
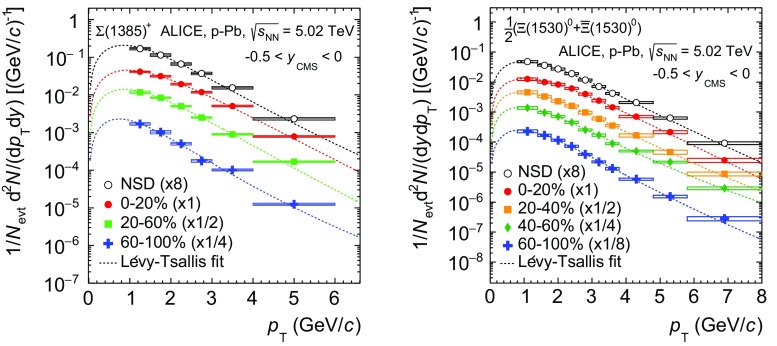
Transverse momentum spectra of $\Sigma ^{*+}$ (*left*) and $\Xi ^{*0}$ (*right*) in different multiplicity classes in the rapidity range $-0.5<y_{\mathrm {CMS}}<0$. For $\Xi ^{*0}$, both particles and antiparticles are analysed together. Statistical (*bars*) and systematic (*boxes*) uncertainties are included. The *dashed curves* are Lévy–Tsallis fit to each individual distribution

The transverse momentum spectra of $\Sigma ^{*+}$ and $\Xi ^{*0}$ in the rapidity range $-0.5<y_{\mathrm {CMS}}<0$ are shown in Fig. 5 for different multiplicity classes and for NSD events. They cover the ranges 1 < $p_{\mathrm{T}}$ < 6 $\mathrm{GeV/c}$ for $\Sigma ^{*+}$ and 0.8 < $p_{\mathrm{T}}$ < 8 $\mathrm{GeV/c}$ for $\Xi ^{*0}$. The spectra obtained for $\overline{\Sigma }^{*-}$, $\Sigma ^{*-}$ and $\overline{\Sigma }^{*+}$ are consistent with the spectrum of $\Sigma ^{*+}$.

The spectra are fitted with a Lévy–Tsallis function [30],

1 $$\begin{aligned} \frac{1}{N_{\mathrm {evt}}}\frac{\mathrm {d}^2N}{\mathrm {d}p_{\mathrm {T}}\mathrm {d}y}= & {} p_{\mathrm {T}} \frac{\mathrm {d}N}{\mathrm {d}y} \frac{(n-1)(n-2)}{nC[nC+m_{0}(n-2)]}\nonumber \\&\times \left[ 1+\frac{ \sqrt{p_{\mathrm {T}}^2+m_{0}^{2}}-m_{0}}{nC}\right] ^{-n}, \end{aligned}$$

where $N_\mathrm {evt}$ is the number of events, $m_{0}$ is the mass of the particle, and *n*, *C* and the integrated yield d$N$/d$y$ are free parameters for the fit. This function was successfully used to describe most of the identified particle spectra in pp collisions  [14, 17, 26].

The values of d$N$/d$y$ and $\,{\langle p_{\mathrm{T}}\rangle }$ shown in Table 6 were calculated by using the experimental spectrum in the measured $p_{\mathrm{T}}$-range and the Lévy–Tsallis fit function outside of the measured $p_{\mathrm{T}}$-range. The contribution from the low-$p_{\mathrm{T}}$ extrapolation to the total d$N$/d$y$ is 36–47% (20–29%) for $\Sigma ^{*+}$ ($\Xi ^{*0}$) moving from low to high multiplicity, while the one from the high-$p_{\mathrm{T}}$ extrapolation is negligible. The systematic uncertainties on d$N$/d$y$ and $\, \langle p_{\mathrm{T}}\rangle$ presented in Table 6 were estimated by repeating the Lévy–Tsallis fit moving randomly (with a Gaussian distribution) the measured points within their $p_{\mathrm{T}}$-dependent systematic uncertainties. The $p_{\mathrm{T}}$-independent uncertainties were further added in quadrature to the systematic uncertainties on d$N$/d$y$. Alternative functional forms, such as Boltzmann–Gibbs Blast-Wave  [31, 32], $m_\mathrm{{T}}$-exponential [32, 33], Boltzmann and Bose–Einstein fit functions were used for both particles to evaluate the systematic uncertainties on the low-$p_{\mathrm{T}}$ extrapolation. The maximum difference between the results obtained with the various fit functions was taken as the uncertainty. These systematic uncertainties, which vary between 5 and 10%, were added in quadrature to the uncertainties for the Lévy–Tsallis fit. The values for $\Sigma ^{*\pm }$ in Table 6 were obtained by averaging those for $\Sigma ^{*+}$, $\overline{\Sigma }^{*-}$, $\Sigma ^{*-}$ and $\overline{\Sigma }^{*+}$ to reduce the statistical uncertainties.

**Table 6:** Integrated yields (d$N$/d$y$) and mean transverse momenta ($\, \langle p_{\mathrm{T}}\rangle$). The values for $\Sigma ^{*\pm }$ are obtained by averaging the values for $\Sigma ^{*+}$, $\overline{\Sigma }^{*-}$, $\Sigma ^{*-}$ and $\overline{\Sigma }^{*+}$. Statistical (first one) and total systematic (second one) uncertainties including the extrapolation from the various fit functions are quoted

Baryon	Multiplicity class	d$N$/d$y$ ($\times$10$^{-3}$)	$\, \langle p_{\mathrm{T}}\rangle$ ($\mathrm{GeV/c}$)
$\Sigma ^{*\pm }$	NSD	49.0 ± 0.6 ± 6.5	1.367 ± 0.009 ± 0.061
	0–20%	90.3 ± 1.4 ± 7.9	1.495 ± 0.012 ± 0.046
	20–60%	52.2 ± 0.8 ± 6.0	1.342 ± 0.010 ± 0.055
	60–100%	15.2 ± 0.4 ± 2.4	1.173 ± 0.015 ± 0.067
1/2($\Xi ^{*0}+\overline{\Xi }^0$)	NSD	12.5 ± 0.3 ± 1.1	1.540 ± 0.016 ± 0.071
	0–20%	27.3 ± 0.6 ± 2.8	1.626 ± 0.016 ± 0.068
	20–40%	17.7 ± 0.5 ± 2.4	1.482 ± 0.020 ± 0.100
	40–60%	10.7 ± 0.3 ± 1.6	1.459 ± 0.025 ± 0.114
	60–100%	3.6 ± 0.1 ± 0.5	1.377 ± 0.023 ± 0.089

### Mean transverse momenta

**Fig. 6:**
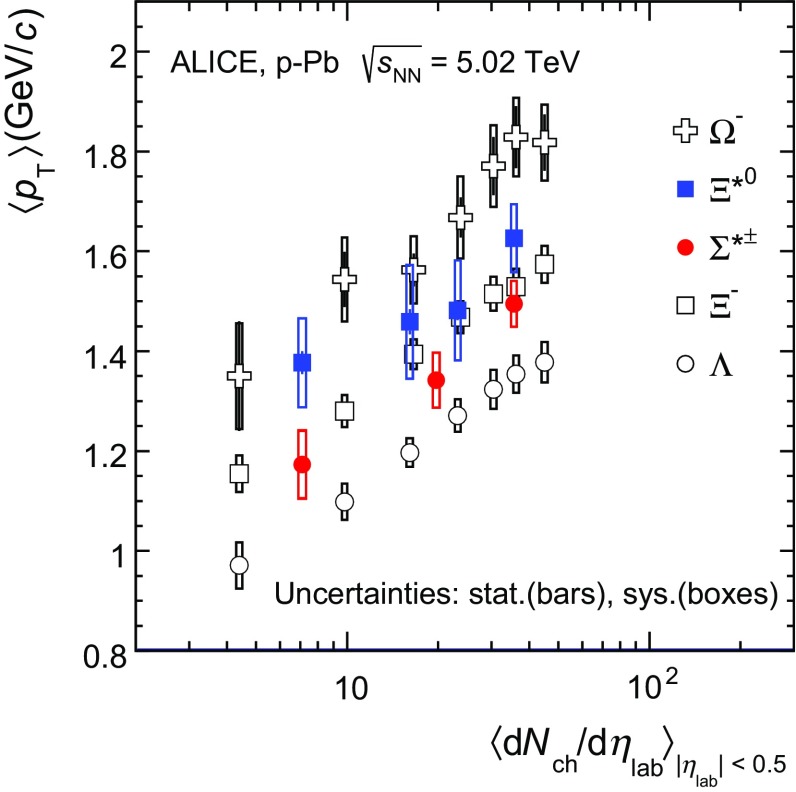
Mean transverse momenta $\, \langle p_{\mathrm{T}}\rangle$ of $\Lambda$, $\Xi ^{-}$, $\Sigma ^{*\pm }$, $\Xi ^{*0}$ and $\Omega ^{-}$ in p–Pb collisions at $\sqrt{s_{\mathrm{NN}}}= 5.02$ TeV as a function of mean charged-particle multiplicity density $\langle$d$N_{\mathrm {ch}}$/d$\eta _{\mathrm {lab}}\rangle$, measured in the pseudorapidity range $\mid \eta _{\mathrm {lab}}\mid<$ 0.5. The results for $\Lambda$, $\Xi ^{-}$ and $\Omega ^{-}$ are taken from [10, 14, 24]. Statistical and systematic uncertainties are represented as *bars* and *boxes*, respectively. The $\Omega ^-$ and $\Xi ^-$ points in the 3rd and 4th lowest multiplicity bins are slightly displaced along the abscissa to avoid superposition with the $\Xi ^{*0}$ points

Figure 6 shows the mean transverse momentum $\, \langle p_{\mathrm{T}}\rangle$ as a function of mean charged-particle multiplicity density $\langle$d$N_{\mathrm {ch}}$/d$\eta _{\mathrm {lab}}\rangle$ at midrapidity. The results for $\Sigma ^{*\pm }$ and $\Xi ^{*0}$ are compared with those for other hyperons observed in p–Pb collisions at $\sqrt{s_\mathrm{NN}}~=~5.02$ TeV [10, 24].

Increasing trends from low to high multiplicities are observed for all hyperons. For both $\Sigma ^{*\pm }$ and $\Xi ^{*0}$, the mean transverse momenta increase by 20% as the mean charged-particle multiplicity increases from 7.1 to 35.6. This result is similar to the one obtained for the other hyperons. Furthermore, a similar increase has been observed also for K$^{\pm }$, K$_\mathrm{{S}}^{0}$, K$^{*}(892)^0$ and $\phi$ [14], whereas protons are subject to a larger ($\sim$33%) increase in the given multiplicity range, as discussed also in Ref. [24].

**Fig. 7:**
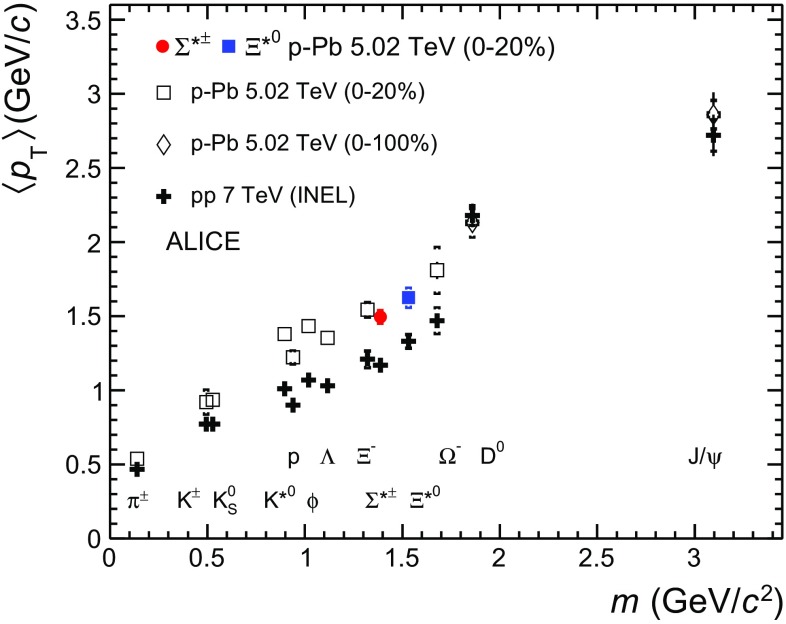
Mass dependence of the mean transverse momenta of identified particles for the 0–20% V0A multiplicity class and with $-0.5<y_{\mathrm {CMS}}<0$ in p–Pb collisions at $\sqrt{s_{\mathrm {NN}}}= 5.02$ TeV [10, 24], and in minimum-bias pp collisions at $\sqrt{s}~=~7$ TeV [17] with $|y_{\mathrm {CMS}}|<0.5$. Additionally, $D^0$ and *J*/$\psi$ results are plotted. The $D^0$ and *J*/$\psi$ were measured in different rapidity ranges: $|y_{\mathrm {CMS}}|<0.5$ [34] ($|y_{\mathrm {CMS}}|<0.9$ [35]) for $D^0$ (*J*/$\psi$) in pp and $-0.96< y_{\mathrm {CMS}}< 0.04$ [34] ($-1.37<y_{\mathrm {CMS}}<0.43$ [36]) for $D^0$ (*J*/$\psi$) in p–Pb. Note also that the results for $D^0$ and *J*/$\psi$ in p–Pb collisions are for the 0–100% multiplicity class

In all multiplicity classes, the $\,{\langle p_{\mathrm{T}}\rangle }$ follows an approximate mass ordering: $\,{\langle p_{\mathrm{T}}\rangle }_{\Lambda }<\,{\langle p_{\mathrm{T}}\rangle }_{\Xi ^-} \simeq \,{\langle p_{\mathrm{T}}\rangle }_{\Sigma ^{*\pm }}<\,{\langle p_{\mathrm{T}}\rangle }_{\Xi ^{*0}}<\,{\langle p_{\mathrm{T}}\rangle }_{\Omega ^{-}}$. The $\,{\langle p_{\mathrm{T}}\rangle }$ of $\Sigma ^{*\pm }$ looks systematically lower than the $\,{\langle p_{\mathrm{T}}\rangle }$ of $\Xi ^{-}$, despite the larger mass of $\Sigma ^{*\pm }$. The uncertainties, however, are too large to draw any conclusion on possible hints of violation of the mass hierarchy. This hierarchy of mass-ordering, also including $D^0$ and *J*/$\psi$ in the comparison, is displayed in Fig. 7. Note, however, that the $D^0$ and *J*/$\psi$ were measured in different rapidity ranges: $|y_{\mathrm {CMS}}|<0.5$ [34] ($|y_{\mathrm {CMS}}|<0.9$ [35]) for $D^0$ (*J*/$\psi$) in pp and $-0.96< y_{\mathrm {CMS}}< 0.04$ [34] ($-1.37<y_{\mathrm {CMS}}<0.43$ [36]) for $D^0$ (*J*/$\, {\psi }$) in p–Pb, and the results for $D^0$ and *J*/$\psi$ in p-Pb collisions are for the 0–100% multiplicity class. This mass dependence is observed in both p–Pb and pp collisions. It was observed also by the STAR collaboration [37] in MB pp, MB d–Au and central Au–Au collisions.

Furthermore, for the light-flavour hadrons, the mean transverse momenta in p–Pb collisions are observed to be consistently higher than those in pp collisions at 7 TeV. The situation for the charm hadrons is different, where $\,{\langle p_{\mathrm{T}}\rangle }$ appears compatible between both colliding systems. The discrepancy is likely due to different production mechanisms for heavy and light flavours and to a harder fragmentation of charm quarks. Specifically, the fact that $\,{\langle p_{\mathrm{T}}\rangle }$ remains similar in pp and in p–Pb is consistent with (i) the fact that p–Pb collisions can be considered as a superposition of independent nucleon-nucleon collisions for what concerns *D*-meson production, as described in [34], and/or (ii) with the effects of shadowing in p–Pb which reduces the production at low $p_{\mathrm{T}}$ and thus increasing the overall $\,{\langle p_{\mathrm{T}}\rangle }$ for *J*/$\psi$ [36]; the small $p_{\mathrm{T}}$ hardening expected in pp when going from 5.02 to 7 TeV is apparently not enough to counter-balance the situation.

Because of small decrease of the $\,{\langle p_{\mathrm{T}}\rangle }$ for proton and $\Lambda$ relative to those for $\, \mathrm{K}^{*0}$ and $\phi$, two different trends for mesons and baryons have been suggested [38]. Even including $D^0$ and *J*/$\psi$, as shown in Fig. 7, a different trend for mesons and baryons cannot be convincingly established.

### Integrated particle ratios

**Fig. 8:**
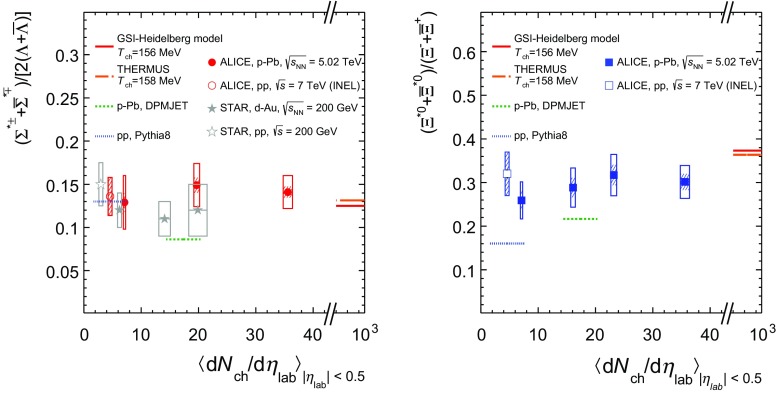
(*Left*) ratio of $\Sigma ^{*\pm }$ to $\Lambda$ and (*Right*) ratio of $\Xi ^{*0}$ to $\Xi ^-$ measured in $\mathrm{pp}$ [17, 32, 37, 39], d–Au [32, 37] and $\text{ p--Pb }$ [10, 24] collisions, as a function of $\langle$d$N_{\mathrm {ch}}$/d$\eta _{\mathrm {lab}}\rangle$ measured at midrapidity. Statistical uncertainties (*bars*) are shown as well as total systematic uncertainties (*hollow boxes*) and systematic uncertainties uncorrelated across multiplicity (*shaded boxes*). A few model predictions are also shown as *lines* at their appropriate abscissa

The integrated yield ratios of excited to ground-state hyperons  [10, 17, 24, 32, 37, 39] with the same strangeness content, for different collision systems and energies, are shown in Fig. 8 as a function of $\langle$d$N_{\mathrm {ch}}$/d$\eta _{\mathrm {lab}}\rangle$. In both cases, the variation of the integrated yield ratio with mean multiplicity is within experimental uncertainties. In fact, the similar flat behaviour of $\Sigma ^{*\pm }/\Lambda$ and $\Xi ^{*0}/\Xi ^-$ is remarkable, when considering their different lifetimes and other properties such as spin and mass.

**Fig. 9:**
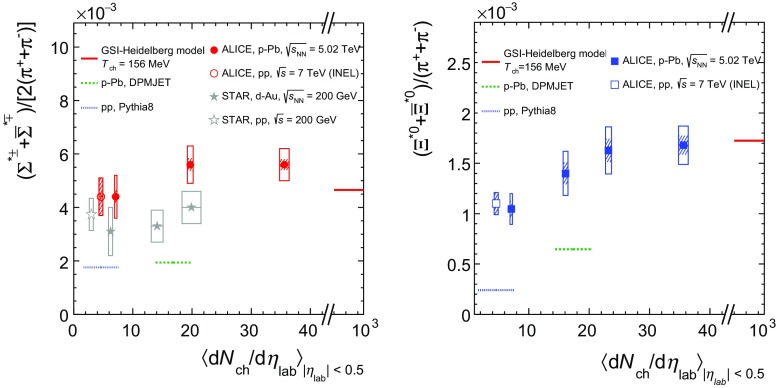
(*Left*) ratio of $\Sigma ^{*\pm }$ to $\pi ^{\pm }$ and (*Right*) ratio of $\Xi ^{*0}$ to $\pi ^{\pm }$, measured in $\mathrm{pp}$ [17, 32, 41, 42], d–Au [32, 37] and $\text{ p--Pb }$ [24] collisions, as a function of the average charged particle density ($\langle$d$N_{\mathrm {ch}}$/d$\eta _{\mathrm {lab}}\rangle$) measured at midrapidity. Statistical uncertainties (*bars*) are shown as well as total systematic uncertainties (*hollow boxes*) and systematic uncertainties uncorrelated across multiplicity (*shaded boxes*). A few model predictions are also shown as *lines* at their appropriate abscissa

The results are compared with model predictions, PYTHIA8 for $\mathrm{pp}$ at 7 TeV [20] and DPMJET for $\text{ p--Pb }$ at 5.02 TeV [19] collisions. The $\Sigma ^{*\pm }$/$\Lambda$ ratios are consistent with the values predicted by PYTHIA8 in $\mathrm{pp}$ collisions, whereas the DPMJET prediction for $\text{ p--Pb }$ collisions is lower than the experimental data. The measured $\Xi ^{*0}$/$\Xi ^-$ ratios appear higher than the corresponding predictions for both systems. Note that the PYTHIA8 [20] and DPMJET [19] values in Figs. 8 and 9 were obtained respectively for INEL pp and NSD p–Pb events, which have corresponding mean charged-particle multiplicities of $\langle$d$N_{\mathrm {ch}}$/d$\eta _{\mathrm {lab}}\rangle _\mathrm {INEL}$ = 4.60 $^{+0.34}_{-0.17}$  [40] and $\langle$d$N_{\mathrm {ch}}$/d$\eta _{\mathrm {lab}}\rangle _\mathrm {NSD}$ = 17.4 ± 0.7  [23]. These predictions are indicated as dotted and dashed lines with arbitrary lengths in the pertinent multiplicity regions in Figs. 8 and 9. Fig. 9 will be discussed later.

The results are also compared to thermal model predictions [7, 18]. For small systems a canonical treatment is a priori required to take into account exact strangeness conservation [18]. This approach leads to a dependence on system size as can be seen in p-Pb collisions studying multi-strange hadrons [10]. For the chosen ratios, however, the canonical corrections are identical for numerator and denominator (same strangeness quantum number). Therefore, the grand canonical values are used in Fig. 8 for two models [7, 18], which are marked at the asymptotic limit, corresponding to the mean charged-particle multiplicity in Pb–Pb [43].

The constant behaviour of the yield ratios of excited to ground-state hyperons with same strangeness content indicates that neither regeneration nor re-scattering dominates with increasing collision system size, even for $\Sigma ^{*\pm }$, which has a shorter lifetime than $\Xi ^{*0}$ by a factor of 4. It is especially interesting to consider the constant behaviour of $\Sigma ^{*\pm }$ /$\Lambda$ ratio in contrast to the apparent decrease observed for $\mathrm{K}^{*0}/\mathrm{K}^{-}$ ratio in the same $\langle$d$N_{\mathrm {ch}}$/d$\eta _{\mathrm {lab}}\rangle$ range [14], in spite of the similarly short lifetimes of $\Sigma ^{*\pm }$ and $\mathrm{K}^{*0}$. In Pb–Pb collisions, both behaviours are predicted by the EPOS3 model [44, 45], which employs the UrQMD model [46] for the description of the hadronic phase. In addition, the $\Sigma ^{*\pm }$/$\Lambda$ ratios at LHC energies turn out to be comparable with the results obtained at lower energies by the STAR collaboration [32, 37].

The integrated yield ratios of excited hyperons to pions are shown in Fig. 9 to study the evolution of relative strangeness production yields with increasing collision system size. Considering the relatively small systematic uncertainties uncorrelated across multiplicity (shaded boxes), one observes increasing patterns by 40–60% relative to results in $\mathrm{pp}$ collisions at the same $\sqrt{s_{\mathrm {NN}}}$, depending on the strangeness contents. These results are consistent with previous observations of ground-state hyperons to pion ratios measured at ALICE [10]. The constant behavior of the $\Sigma ^{*\pm }$/$\Lambda$ and $\Xi ^{*0}/\Xi ^-$ ratios indicates that the strangeness enhancement observed in p-Pb collisions depends predominantly on the strangeness content, rather than on the hyperon mass. Results from low-energy collisions [32, 37, 42] show a similar pattern in spite of the narrower range accessible for mean charged-particle multiplicity. In both cases, QCD-inspired predictions like PYTHIA for pp [20] and DPMJET for p–Pb [19] clearly underestimate the observed yield ratios, while the statistical one seems to be comparable with results from high multiplicity events.

## Conclusions

Transverse momentum spectra of $\Sigma ^{*\pm }$ and $\Xi ^{*0}$ produced in $\text{ p--Pb }$ collisions at $\sqrt{s_\mathrm{NN}}$ = 5.02 TeV have been measured, and the yields and mean $p_{\mathrm{T}}$ values have been extracted with the help of Lévy–Tsallis fits. The mean $p_{\mathrm{T}}$ of these hyperon resonances exhibit a similarly increasing pattern as other hyperons ($\Lambda$, $\Xi ^-$, $\Omega ^-$), depending on mean multiplicity and following the approximate mass ordering observed for other particles despite of relatively large uncertainties. The integrated yield ratios of excited to ground-state hyperons, with the same strangeness content, show a flat behaviour over the whole mean multiplicity range. The $\Sigma ^{*\pm }$/$\Lambda$ ratio does not show a variation with collision energy, nor with increasing system size. The $\Xi ^{*0}$/$\Xi ^-$ ratios are higher than predicted by event generators. Both ratios agree with thermal model values. The yield ratios relative to pions show a gradual increase with $\langle$d$N_{\mathrm {ch}}$/d$\eta _{\mathrm {lab}}\rangle$. This rise is consistent with the results of ground-state hyperons produced in the same collision system, i.e. they show a gradual evolution with the system size depending only on the strangeness content.

The current measurement represents a relevant baseline for further investigation in Pb–Pb collisions. It will be especially valuable to compare the $\Sigma ^{*\pm }$/$\Lambda$ ratio with $\mathrm{K}^{*0}/\mathrm{K}^{-}$, since $\Sigma ^{*\pm }$ and $\mathrm{K}^{*0}$ have similar lifetimes. A complete set of such measurements for many resonances ($\rho$, $\mathrm{K}^{*0}$, $\phi$, $\Sigma ^{*\pm }$, $\Lambda ^*$, $\Xi ^{*0}$) with different lifetimes will allow the properties of the hadronic phase to be studied in more detail.

## Footnotes

1 Similarly to what has been observed in the pp analysis [17], the distributions of $\Sigma ^{*-}$ ($\overline{\Sigma }^{*+}$), not shown in this paper, have an additional peak at $\sim$1.321 $\mathrm{GeV/c}^{2}$, as narrow as $\sim$3 MeV/c$^{2}$, due to the residual $\Xi (1321)^{-}$ ($\overline{\Xi }(1321)^{+}$), escaping the filter on the DCA of $\Lambda$ to PV mentioned above.
